# Sliding hiatal hernia detected by transnasal esophagogastroscopy is associated with poorer treatment response in reflux-associated globus sensation

**DOI:** 10.1038/s41598-026-65138-8

**Published:** 2026-08-12

**Authors:** Jiri Podzimek, Peter Jecker, Orlando Guntinas-Lichius

**Affiliations:** 1 Department of Otorhinolaryngology, Plastic Head and Neck Surgery, Klinikum Bad Salzungen GmbH, Bad Salzungen, Germany; 2 Department of Otorhinolaryngology, Jena University Hospital, Jena, Germany

**Keywords:** Globus sensation, Reflux disease, Gastroesophageal junction, Transnasal esophagogastroscopy, Treatment response, Diseases, Gastroenterology, Medical research

## Abstract

Transnasal esophagogastroscopy (TNE) may provide clinically relevant information on gastroesophageal junction morphology in patients with globus sensation and reflux disease. This prospective single-arm interventional cohort study with a pre-post design evaluated the association between TNE findings and treatment response in patients with objectively confirmed reflux disease. Ninety-two patients with globus sensation underwent dual-probe pH monitoring; reflux disease was detected in 59 patients. These patients underwent TNE and completed the German version of the Glasgow Edinburgh Throat Scale (GETS-G) before and after 12 weeks of antireflux treatment. Pathological TNE findings were identified in 25 patients, with sliding hiatal hernia being the most frequent abnormality (18 patients). The mean GETS-G score decreased significantly from 38.5 ± 7.1 to 24.0 ± 8.1 after treatment. Using the ≥ 20% GETS-G reduction threshold, 48 of 59 patients met the study-defined response criterion. Sliding hiatal hernia was not significantly associated with reflux subtype but was independently associated with poorer symptom improvement. Hill grades I–II were associated with greater improvement than Hill grades III–IV. Endoscopic assessment of hiatal hernia and Hill classification may support individualized management of reflux-associated globus sensation.

## Introduction

Globus sensation is a frequent symptom in otolaryngological practice and is typically described as a persistent or intermittent non-painful sensation of a lump, foreign body, or tightness in the throat^1,2^. Given its heterogeneous etiology, a differentiated diagnostic and therapeutic approach based on the individual clinical findings has been proposed^3^. Although the symptom is benign in most patients, it often causes considerable anxiety and may lead to repeated medical consultations^4,5^. The etiology of globus sensation is multifactorial and may include laryngopharyngeal reflux disease, gastroesophageal reflux disease, esophageal motility disorders, psychological factors, upper and lower esophageal sphincter dysfunction, thyroid disease, and structural lesions of the pharynx or esophagus^6,7^.

Reflux disease is commonly considered one of the most relevant potential contributors to globus symptoms^8,9^. Current guidelines emphasize that laryngeal or pharyngeal symptoms alone are insufficient to establish the diagnosis of reflux disease and that objective reflux testing should be considered, particularly in patients without typical symptoms or in those with insufficient response to empirical therapy^10,11^.

Hiatal hernia may represent an important anatomical factor in patients with reflux disease^12^. By separating the lower esophageal sphincter from the diaphragmatic crura, a hiatal hernia can impair the antireflux barrier, increase esophageal acid exposure, and promote reflux events^13,14^. In addition, the competence of the gastroesophageal flap valve, commonly assessed using the Hill classification during retroflexed endoscopy, may provide further information regarding the integrity of the gastroesophageal junction^15^. However, the clinical relevance of hiatal hernia and Hill grade in patients presenting with globus sensation remains insufficiently defined^16^.

Medical treatment with proton pump inhibitors and lifestyle modifications remain a common first-line therapeutic approach in patients with documented reflux disease. Nevertheless, therapeutic response in patients with throat symptoms is variable. Large randomized data have shown no clear benefit of PPI therapy over placebo in unselected patients with persistent throat symptoms, underlining the need for better phenotyping and objective diagnostic work-up^17,18^.

Transnasal esophagogastroscopy (TNE) is a useful outpatient diagnostic tool in otolaryngology for patients with globus sensation and suspected reflux disease^19^. Compared with conventional sedated gastroscopy, TNE is usually performed without sedation and is well tolerated, while still allowing visualization of the esophagus, gastroesophageal junction, and stomach, including reflux-related mucosal changes and anatomical findings such as axial hiatal hernia^20,21^.

To our knowledge, evidence on the association between hiatal hernia size, gastroesophageal flap valve morphology, and reflux-associated globus sensation remains limited. The aim of the present study was to evaluate TNE findings in patients with globus sensation and objectively proven reflux disease, focusing on sliding hiatal hernia and the Hill classification^22^. In addition, we investigated whether hiatal hernia and gastroesophageal flap valve morphology influenced symptom improvement after standardized antireflux therapy.

## Methods

### Design and setting

A prospective single-arm interventional cohort study with a pre-post outcome was carried out at the Department of Otorhinolaryngology of Bad Salzungen Hospital, Germany, between June 2021 and December 2024. The protocol was reviewed and approved by the Institutional Review Board of the Bad Salzungen Hospital and adhered to the principles of the Declaration of Helsinki, Good Clinical Practice, and applicable regulatory requirements. All participants provided written informed consent before inclusion.

**Fig. 1:**
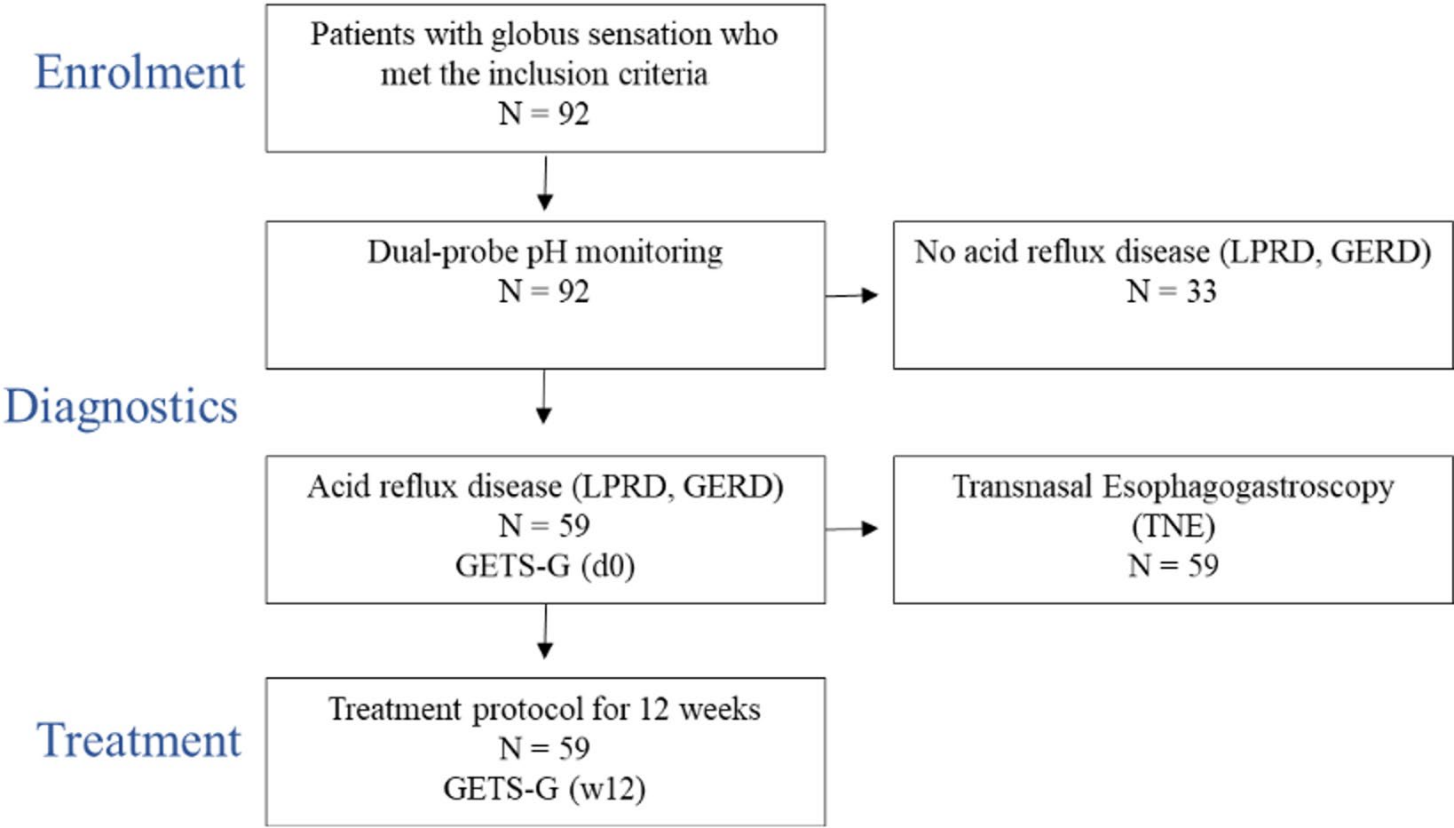
Flow chart of the study population; d, day; w, week; GETS-G, German version of the Glasgow Edinburgh Throat Scale; LPRD, laryngopharyngeal reflux disease; GERD, gastroesophageal reflux disease.

Eligible participants were adults aged 18 years or older who presented with globus sensation persisting for a minimum of 3 months. Overall, 92 patients were enrolled. Exclusion criteria, as recently reported, comprised treatment with proton pump inhibitors or histamine H2-receptor antagonists within the 6 months preceding enrolment, previous surgery involving the upper aerodigestive tract, tobacco use on at least 5 days per week, alcohol consumption on at least 1 day per week, asthma, head and neck tumors or cystic lesions, and prior radiotherapy to the cervical or head and neck region^23^. An overview of the study workflow is provided in Fig. 1.

### Ambulatory dual-probe pH monitoring

Ambulatory 24-hour dual-probe pH monitoring (Medtronic, Meerbusch, Germany) was performed to comprehensively assess acid exposure in the distal esophagus and hypopharynx. The acid exposure time (AET) and reflux area index (RAI), identified as the two most sensitive parameters, were calculated from the hypopharyngeal probe measurements, whereas the DeMeester score was derived from the distal esophageal probe data. Thresholds indicating clinically significant reflux were defined as an AET exceeding 0.2% and an RAI greater than 6.8 for laryngopharyngeal reflux disease (LPRD)^24^, and a DeMeester score above 14.72 for gastroesophageal reflux disease (GERD)^25^.

### Transnasal esophagogastroscopy (TNE)

**Fig. 2:**
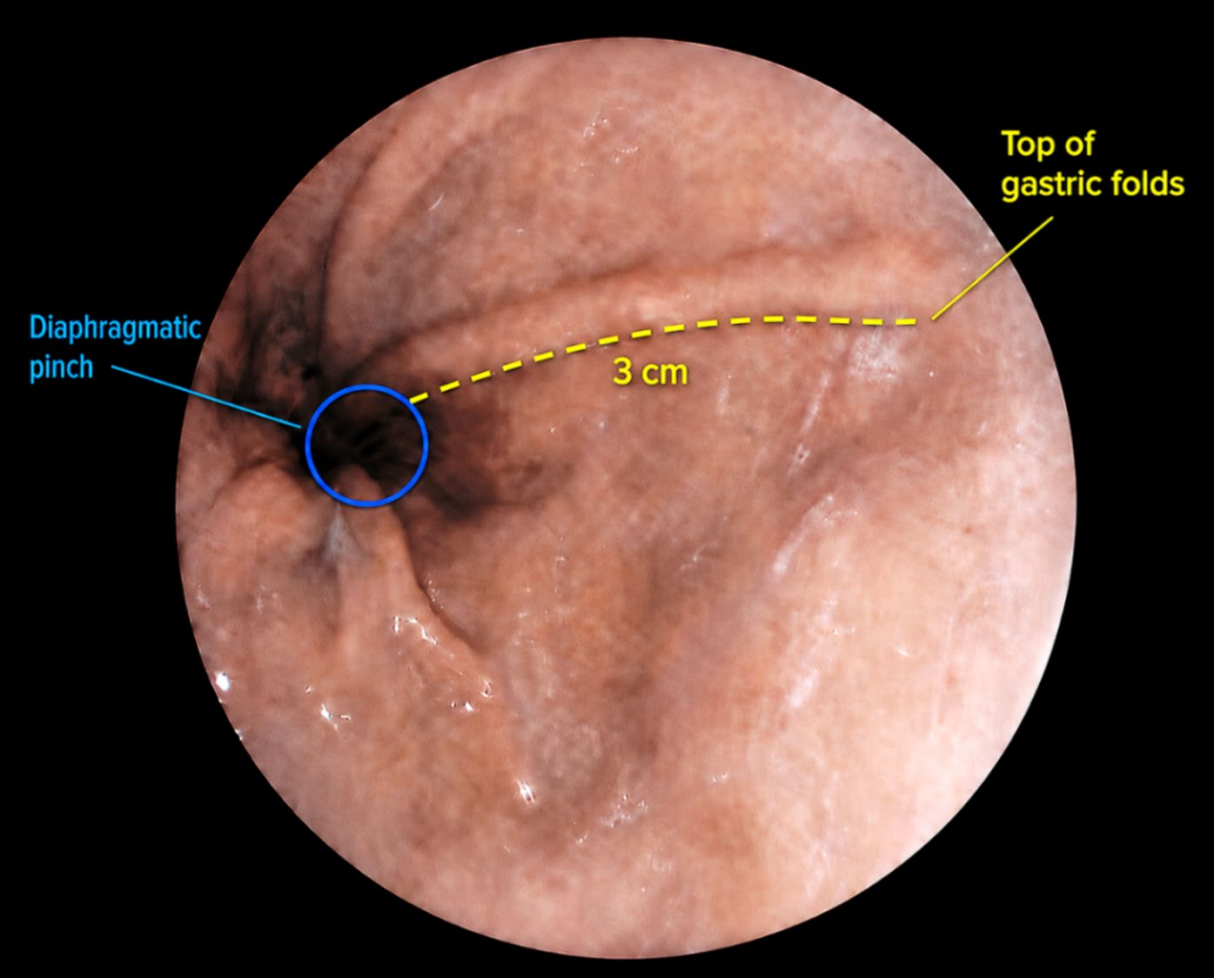
Endoscopic view of a sliding hiatal hernia. Hiatal hernia was defined as a distance of ≥ 2 cm between the diaphragmatic pinch and the proximal end of the gastric folds.

The examination was performed with the patient seated and unsedated after appropriate informed consent was provided (Diomed-Form Endo 4, Thieme Compliance, Erlangen, Germany). The nasal mucosa was decongested with 0.1% xylometazoline nasal spray. Local anesthesia of the nasal mucosa and pharynx was achieved using 10% lidocaine spray. The examination was performed using a gastroscope with a diameter of 4.8 mm (EG-530NP; Fujinon, Saitama, Japan). The tip of the gastroscope was coated with lubricant (xylocaine gel 2%). The endoscope was advanced through the nose and nasopharynx to the larynx, and the mucosa of the pharynx and larynx was assessed. The endoscope was positioned in the piriform recess, and the patient was asked to swallow a small amount of water. During swallowing, the cricopharyngeal muscle relaxed, and the endoscope was advanced effortlessly through the upper esophageal sphincter into the esophagus. The endoscope was then advanced with air insufflation to the lower esophageal sphincter and squamocolumnar junction. This facilitated the evaluation of the esophageal mucosa and the identification of potential indicators of reflux, such as esophagitis. Furthermore, during antegrade endoscopy, the distance between the gastroesophageal junction and diaphragmatic hiatus was measured. A sliding hiatal hernia was diagnosed when the gastroesophageal junction, usually identified by the proximal extent of the gastric folds, is separated from the diaphragmatic aperture by ≥ 2 cm (Fig. 2)^26^. One principal pathological TNE finding was recorded for each patient.

**Fig. 3:**
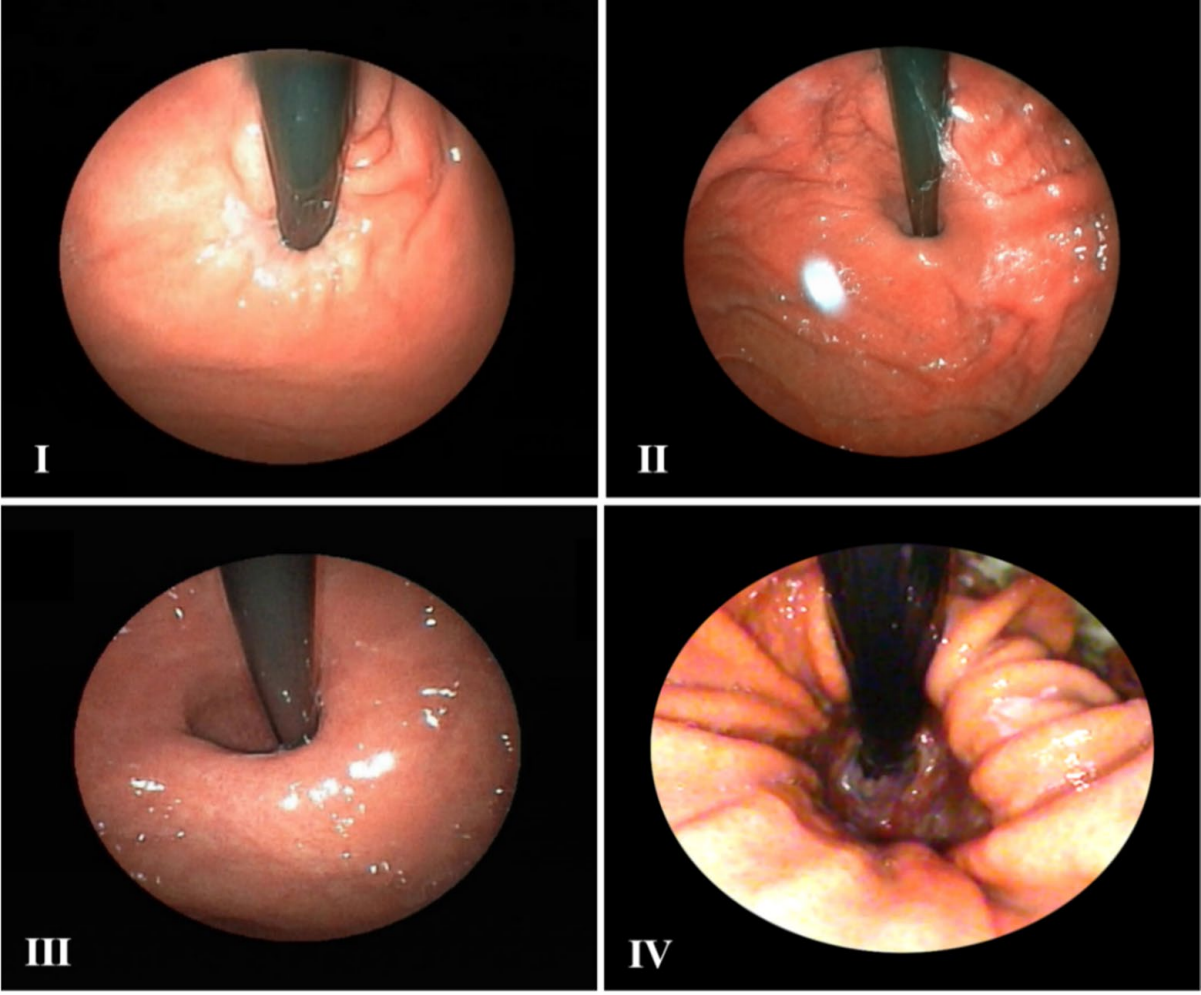
Endoscopic classification of gastroesophageal junction – Hill grades I-IV.

After the endoscope entered the stomach, the hollow organ was inflated with air. The stomach contents were suctioned to examine the entire gastric mucosa. Subsequently, the endoscope was inverted (retroflexion) to assess the gastroesophageal flap valve according to the Hill classification (Table 1; Fig. 3). To allow for a precise evaluation of the esophageal mucosa over the entire circumference, the esophagus was distended with air insufflation while the endoscope was withdrawn. During withdrawal, the entire esophageal mucosa, upper esophageal sphincter, and hypopharynx were reassessed.

**Table 1:** Hill classification.

Hill	Endoscopic assessment of the gastroesophageal flap valve
Grade I	Prominent fold of tissue is present, with tight closure around the endoscope
Grade II	Fold is less prominent; closure around the endoscope is less tight and can be transiently open during respiration
Grade III	Fold is barely present, and the tissue does not close around the endoscope
Grade IV	Fold is absent; the esophagogastric junction is wide open, with a noticeable hiatal hernia

### Questionnaire

All participants with proven reflux disease were asked to complete the GETS-G to establish a pretreatment baseline (d0) for symptom severity and to assess treatment response after 3 months (w12). The GETS-G comprises 12 items covering globus sensation, dysphagia, chronic pharyngeal irritation, and symptom-related distress. Each item is scored from 0 to 7, yielding a total score ranging from 0 to 84, with higher scores indicating greater symptom severity and distress. Treatment response was defined as a reduction of ≥ 20% in the GETS-G score. The percentage score improvement was calculated as follows: [(baseline score − post-treatment score)/baseline score] × 100.

### Therapy

The treatment protocol was based on the recommendations of young otolaryngologists of the International Federation of ORL Societies^27^. Patients with proven LPRD and/or GERD after pH monitoring received advice on a low-fat, low-acid diet and lifestyle modifications and were treated with a proton pump inhibitor (pantoprazole 20 mg twice daily at 12-hour intervals before meals) for 3 months.

### Statistics

Statistical analyses were performed using SPSS software, version 28.0.0.0 (IBM Deutschland GmbH, Ehningen, Germany). Continuous variables were expressed as mean ± standard deviation, while categorical variables were expressed as absolute numbers and percentages. The Mann-Whitney U test was used to compare the scaled data of two independent subgroups. The Wilcoxon test was conducted to analyze data between the two dependent groups. The association between hiatal hernia and reflux category and sex was assessed using crosstabs and Pearson’s chi-square test. The effect size was reported as Cramér’s V. The impact of hiatal hernia and Hill classification on treatment response was assessed using linear regression analysis. The threshold for statistical significance was established at *p* ≤ 0.05.

## Results

### Patient characteristics

The characteristics of the study patients are shown in Table 2. A total of 92 participants were enrolled in this study. The mean age of the study population was 59 ± 6.9 years. The sample included 39 male (42.4%) and 53 female (57.6%) patients. No significant age differences were observed between patients with and without reflux disease. All patients successfully underwent dual-probe pH monitoring without experiencing any complications. Among these individuals, 59 were diagnosed with reflux disease. These 59 patients (59.3% female, median age: 61 years) constituted the study sample for further analysis. The types of reflux are summarized in Table 2. All 59 patients with proven reflux underwent TNE, and full-spectrum endoscopy was successfully performed in each case without any complications.

**Table 2:** Patient characteristics, LPRD, laryngopharyngeal reflux disease; GERD, gastroesophageal reflux disease.

Parameter	Frequency (*N*)	%
All	92	100
**Gender**		
Male	39	42.4
Female	53	57.6
**pH monitoring**		
All	92	100
Positive	59	64.1
Negative	33	35.9
**Type of reflux**		
All patients with objectified reflux	59	100
LPRD	19	32.2
LPRD + GERD	27	45.8
GERD	13	22.0
**Transnasal esophagogastroscopy (principal findings)**		
All patients with objectified reflux	59	100
Axial hiatal hernia	18	30.5
Erosive esophagitis	5	8.5
Tumor	2	3.4
Heterotopic gastric mucosa	0	0
No finding	34	57.6
**Hill classification**		
All patients with objectified reflux	59	100
Grade I	14	23.7
Grade II	20	33.9
Grade III	17	28.8
Grade IV	8	13.6

### Endoscopic findings

Pathological alterations were identified in 25 patients. The most prevalent finding was sliding hiatal hernia, which was observed in 18 individuals. The mean hiatal hernia length was 26.1 ± 7.4 mm, with a range of 20–45 mm. There was no significant difference in hernia length according to the type of reflux or the Hill grade, respectively (*p* = 0.668; *p* = 0.153). Erosive esophagitis was detected in five cases. A tumor was identified in two patients. In one instance, a benign hemangioma was suspected, whereas in the other, adenocarcinoma of the distal esophagus was confirmed histologically.

### GETS-G and treatment outcome

The mean GETS-G score at d0 for patients with reflux disease was 38.5 ± 7.1, with scores ranging from 24 to 59. The detailed characteristics of the GETS-G score are presented in Table 3. Following treatment (w12), the GETS-G score decreased to 24.0 ± 8.1, with a range of 9–44, indicating a statistically significant within-group improvement (*p* < 0.001). Using the prespecified ≥ 20% reduction threshold, 48 of 59 patients (81.4%) met the study-defined response criterion. The mean percentage reduction in the GETS-G score in the total reflux cohort was 37.6 ± 15.6%, with a range from 12.0% to 80.0%. Among patients meeting the study-defined response threshold, the mean percentage reduction was 42.2 ± 13.3%, whereas the remaining patients showed a mean reduction of 16.6 ± 2.2%.

**Table 3:** GETS-G scores related to patients’ characteristics (*n* = 59 with reflux), GETS-G, German version of the Glasgow Edinburgh Throat Scale; LPRD, laryngopharyngeal reflux disease; GERD, gastroesophageal reflux disease.

Parameter	Mean ± SD	*p*
**Sex**		
Male	41 ± 7	0.210
Female	36.8 ± 6.7	
**Type of reflux**		
LPRD	40 ± 8	0.833
LPRD + GERD	38.8 ± 6.9	
GERD	37.4 ± 6.5	
**Transnasal esophagogastroscopy**		
Axial hiatal hernia (yes)	41.3 ± 7.8	0.580
Axial hiatal hernia (no)	37.3 ± 6.5	
Erosive esophagitis (yes)	39.1 ± 7.1	0.770
Erosive esophagitis (no)	38.4 ± 7.2	
**Hill classification**		
Grade I	38.5 ± 5.3	0.473
Grade II	36.8 ± 7.3	
Grade III	38.8 ± 6.2	
Grade IV	42.4 ± 10.4	
Grade I + II	37.5 ± 6.5	0.216
Grade III + IV	39.9 ± 7.7	

### Hiatal hernia and sex

Hiatal hernia was present in 14 of 35 women (40%) and in 4 of 24 men (16.7%). No statistically significant association was found between sex and the presence of hiatal hernia (Pearson’s chi-square test: χ²(1) = 3.66, *p* = 0.056). The effect size indicated a small-to-moderate association (Phi = 0.249; Cramér’s V = 0.249). Fisher’s exact test was not statistically significant (*p* = 0.084), although the results suggested a borderline trend.

### Hiatal hernia and type of reflux

No statistically significant association was found between hiatal hernia and reflux category (Pearson’s chi-square test: χ²(2) = 0.73, *p* = 0.695). The effect size was small (Cramér’s V = 0.11). One of six cells (16.7%) had an expected count below 5, with a minimum expected count of 4.41, indicating that the assumptions for the Pearson chi-square test were acceptably met.

### Impact of hiatal hernia on therapeutic outcome

In the linear regression analysis, the presence of hiatal hernia was significantly associated with a poorer treatment response (B = −5.16, 95% CI: −8.02 to −2.31, *P* = 0.001). The baseline GETS-G score was included as a covariate to adjust for initial symptom severity. After adjusting for the baseline GETS-G score, patients with hiatal hernia showed an approximately 5-point smaller improvement in GETS-G score compared with patients without hiatal hernia. The GETS-G change data are presented in Table 4. The baseline GETS-G score was not significantly associated with treatment response (B = −0.046, *P* = 0.636). The overall model was significant (F(2,56) = 7.50, *p* = 0.001) and explained 21.1% of the variance in score change (R² = 0.211). However, because only 18 patients had a hiatal hernia, this estimate should be considered exploratory and hypothesis-generating.

**Table 4:** GETS-G change data based on hiatal hernia and Hill group, d, day; w, week; GETS-G, German version of the Glasgow Edinburgh Throat Scale; SD, standard deviation.

Group	n	Baseline GETS-G (d0) Mean ± SD	Post treatment GETS-G (w12) Mean ± SD	Absolute GETS-G change Mean ± SD	Percentage improvement Mean ± SD
**Hiatel hernia**					
Absent	41	37.3 ± 6.5	21.2 ± 6.9	16.1 ± 7.8	41.4 ± 15.5%
Present	18	41.3 ± 7.8	30.3 ± 7.3	11.0 ± 5.1	29.1 ± 12.4%
**Hill Group**					
Grades I-II	34	37.5 ± 6.5	21.1 ± 6.9	16.4 ± 4.6	42.5 ± 14.0%
Grades III-IV	25	39.9 ± 7.7	27.9 ± 8.2	12.0 ± 9.6	31.0 ± 15.5%
**Total cohart**	**59**	**38.5 ± 7.1**	**24.0 ± 8.1**	**14.5 ± 7.4**	**37.6 ± 15.6%**

### Influence of Hill classification on therapeutic outcome

In the linear regression analysis, the dichotomized Hill group (Hill grades III-IV = 0; Hill grades I-II = 1) was significantly associated with change in the GETS-G score (B = 4.4, 95% CI: 1.54 to 7.02, *p* = 0.003). Compared with patients with Hill grades 3–4, those with Hill grades 1–2 showed a 4.4-point greater improvement in GETS-G score.

## Discussion

The present study investigated the role of TNE in 59 patients with globus sensation and objectively confirmed reflux disease, with particular emphasis on the gastroesophageal junction and its effects on treatment outcomes. The main findings were that principal pathological endoscopic findings were detected in a substantial proportion of patients (42.4%), with sliding hiatal hernia representing the most frequent abnormality (30.5%). There is limited recent literature on the endoscopic diagnosis of hiatal hernia in patients with globus sensation, irrespective of cause, with reported rates ranging from 6% to 52%^28,29,30^. Similarly, heterogeneous data have been reported for GERD and LPRD^20,31^.

Although hiatal hernia was not significantly associated with reflux subtype, its presence was independently associated with poorer improvement after standardized antireflux treatment, based on GETS-G score. Given the modest cohort size, this association should be regarded as hypothesis-generating and requires confirmation in larger studies. In addition, patients with favorable Hill grades I–II demonstrated significantly greater improvement than those with Hill grades III–IV. This supports the assumption that high-grade impairment of flap valve competence is associated with reduced response to conservative treatment^32^.

The Hill classification appears to be particularly useful in this context^33^. While the presence of a hiatal hernia reflects a measurable anatomical displacement of the gastroesophageal junction, the Hill grade provides a functional-endoscopic assessment of the gastroesophageal flap valve. Previous studies and meta-analytic data have shown that abnormal Hill grades, particularly grades III–IV, are associated with symptomatic GERD and erosive esophagitis^12,15,34^. These results suggest that the anatomical configuration of the gastroesophageal junction may influence therapeutic outcome in patients with globus sensation and proven reflux disease. Therefore, documenting the Hill grade during transnasal esophagogastroscopy may help identify patients in whom reflux symptoms are more strongly driven by anatomical barrier dysfunction. However, Hill grading focuses primarily on flap valve appearance and remains partly subjective. The AFS (American Foregut Society) classification was developed as a more comprehensive alternative that expands Hill grading by incorporating axial hernia length, hiatal aperture diameter, and flap valve status (LDF components), together with a standardized endoscopic methodology^35,36^.

The present findings are clinically relevant because empirical PPI therapy is frequently used in patients with suspected LPRD or reflux-related throat symptoms^37^. However, the therapeutic benefit of PPIs for persistent throat symptoms is controversial. The TOPPITS trial demonstrated no significant advantage of lansoprazole over placebo in patients with persistent throat symptoms, suggesting that symptom-based diagnosis alone may lead to overtreatment^18^. The present study included only patients with objective evidence of reflux disease on dual-probe pH monitoring, which may have contributed to the observed within-group improvement^38^.

The pantoprazole regimen used in this study had relatively low acid-suppressive potency when expressed in omeprazole equivalents. Graham and Tansel estimated the relative potency of pantoprazole as 0.23 compared with omeprazole, corresponding to approximately 4.6 mg of omeprazole equivalents for each 20-mg pantoprazole dose^39^. Accordingly, the observed symptom improvement may also reflect placebo response, natural symptom fluctuation, and the concomitant dietary and lifestyle interventions.

Interestingly, hiatal hernia was not significantly associated with reflux subtype in this cohort. This finding suggests that the presence of a hiatal hernia does not necessarily determine whether patients present with LPRD, GERD, or both. Rather, it may influence reflux burden and treatment response across different reflux phenotypes. This distinction is important because extraesophageal reflux symptoms are often heterogeneous and may not correlate directly with conventional GERD findings. Current literature emphasizes that LPR symptoms are nonspecific, overlap with several non-reflux conditions, and require careful phenotyping and objective testing^11,40,41^.

In the present study, TNE proved to be feasible and safe. All patients with proven reflux disease underwent full-spectrum endoscopic evaluation without complications. This supports the use of TNE as a valuable diagnostic tool in ENT practice. Previous studies have shown that office-based or outpatient TNE is well tolerated and useful in the evaluation of patients with globus sensation, dysphagia, or reflux-related symptoms^19,42,43^. In addition to detecting hiatal hernia and assessing Hill grade, TNE allows visualization of the esophageal mucosa and may reveal clinically relevant findings such as erosive esophagitis and premalignant or neoplastic lesions. In the present cohort, erosive esophagitis was detected in five patients and esophageal tumors were identified in two patients, including one histologically confirmed adenocarcinoma. These findings underline that TNE may provide clinically meaningful information beyond reflux diagnosis alone.

Current GERD guidelines emphasize that endoscopy is useful for identifying complications such as erosive esophagitis, Barrett’s esophagus, strictures, and structural lesions, whereas endoscopy alone cannot reliably confirm or exclude reflux as the cause of extraesophageal symptoms^9^. Therefore, the diagnostic value of TNE in this setting should be interpreted as complementary rather than substitutive to provide additional anatomical and mucosal information. This combined approach with dual-probe pH monitoring may be particularly useful in patients with persistent globus sensation, suspected reflux disease, or insufficient response to empirical treatment.

The therapeutic implications of hiatal hernia should also be considered. In patients with small sliding hiatal hernias, lifestyle modifications and optimized medical treatment may be sufficient. However, in patients with larger hernias, severe flap valve insufficiency, persistent regurgitation, or PPI-refractory symptoms, the underlying problem may be predominantly mechanical and may require closer follow-up and, in selected cases, further gastroenterological or surgical evaluation. Current surgical guidelines emphasize individualized decision-making based on symptoms, hernia type, objective reflux evidence, and patient preference^44^.

Several limitations must be acknowledged. First, this was a single-center uncontrolled study with a relatively small sample size. Second, the prespecified ≥ 20% GETS-G reduction represented an operational study threshold. Therefore, the responder proportion of 81.4% should be interpreted as a descriptive outcome measure rather than as evidence of treatment-specific efficacy. Third, the study focused on acid reflux parameters; weakly acidic and non-acid reflux events were not assessed^45,46^. Finally, the endoscopic assessment of hiatal hernia and Hill grade may be influenced by insufflation, patient position, and the dynamic anatomy of the gastroesophageal junction.

## Conclusions

In patients with globus sensation and objectively confirmed reflux disease, transnasal esophagogastroscopy provides relevant additional information regarding mucosal pathology, sliding hiatal hernia, and gastroesophageal flap valve morphology. Although hiatal hernia was not associated with reflux subtype, it was significantly associated with poorer symptom improvement after standardized antireflux therapy. In addition, favorable Hill grades I–II were associated with better treatment response than Hill grades III–IV. These exploratory findings suggest that endoscopic assessment of the gastroesophageal junction may contribute to treatment stratification in patients with reflux-associated globus sensation, but they require confirmation in larger controlled studies.

## Data Availability

The datasets used and analyzed during the current study are available from the corresponding author upon reasonable request.
